# The New Journal—Its Contents, Etc.

**Published:** 1884-06

**Authors:** E. F. Starr

**Affiliations:** Nacoochee, Ga.


### Correspondence.

### THE NEW JOURNAL—ITS CONTENTS, ETC.

EDITORS JOURNAL :—I have been pleased to see this new issue under the old name with its improved appearance, with its portrait decoration and with its efficient corps of editors, through which "at once it promises what at once it gives." The portrait of Dr. Long is but a just tribute, well placed, to a beneficent discoverer made illustrious by his work.

The contributions are interesting and instructive. The subject of Dr. Gaston's paper is grave and important, and he has well illustrated the propriety and shown the value of complete anæsthesia in such cases as he speaks of, that is, for the purpose of making practicable the correction of unfavorable face presentations. It was well to call the attention of the profession to this method of dealing with these formidable cases, and to illustrate it with such apropos cases in practice. The reassertion of a discovered truth, or of a practical fact in medicine, is sometimes of almost as great importance as the first discovery. For the want of general publicity and universal assertion, many valuable discoveries, ideas and facts have been allowed to lie dormant, hidden or unknown to the general world until they have become a lost part of the common estate of the world's knowledge. So, although Dr. Gaston's views and points in practice are not exactly new, still they are of sufficient importance to be reasserted in the face of those who have not seen the same facts expressed elsewhere. In Leishman's Midwifery, American edition, 1879, the annotator, Dr. Parry, says : "If rotation cannot be effected by the measures recommended by the author, it is doubtful whether the obstruction should 'be called insurmountable,' and the perforator resorted to without a trial of another measure.

In November, 1873, I read a paper before the Obstetrical Society of Philadelphia on the use of the hand to correct unfavorable presentations and positions of the head during labor. In this was related a case of face presentation with the chin behind and to the right side * * . The face was almost at the inferior strait. All attempts to flex the head, to rotate the chin in front, or to deliver by traction with the forceps failed. The woman was completely exhausted, and there seemed to be no alternative but to perform craniotomy. Before resorting to this, however, I passed my whole hand into the pelvis and placed the thumb over the brow and the fingers over the superior maxillary bone, and, pushing forcibly upwards, the head was easily raised above the brim of the pelvis. It was then flexed without any difficulty, and a mento-posterior of the face was converted into an occipito anterior of the vertex * *. To successfully perform this manipulation the woman should be completely under the influence of an anæsthetic. When the patient is thoroughly relaxed by ether it is very surprising what can be done by forcibly pushing the head upwards. Not only does the child ascend, but if the lower segment of the uterus has been carried with the head into the cavity of the pelvis, it may be lifted with its contents above the pelvic brim, when the latter becomes movable and easily manipulated.

Dr. Gaston's cases will serve to confirm Dr. Parry's statements, and help to establish a rule of practice. Dr. Gaston says: "It is not claimed that a rule can be established upon the limited observation of the result in two cases, but such an effect has not, within my range of reading, been noted previously, and a knowledge of this fact may open the way to important steps in correcting all malpositions of the child accompanied with clonic uterine contractions."

"What of Bismuth?" is an interesting and suggestive article by Prof. Miller upon a subject that ought to be driven to a conclusion and upon a question that should be forced to a settlement.

*Cui bono?* What good is there in bismuth? If we decide by the prevailing usage or practice of the profession at the present time we judge, much; if by our own experience we answer, some. But the how and wherefore are terse questions. The Doctor has given a succinct and lucid history of this agent from its introduction, and he has given the negative facts in regard to its inertness as a medicine; among them are these: "The most careful observation has detected no impression whatever upon the nervous system, or the slightest change in its delicate phenomena;" and "it neither increases, diminishes nor alters the secretions of any gland or emunctory of the body."

Now I remember one case, at least, of a man suffering with frequent and irregular pains and uneasiness in the left hypochondric and iliac regions, with frequent small piddling stools, (indicating either hyperæsthesia of the intestinal canal or of subacute inflammation of that organ,) for which I prescribed five to ten grain doses of the subnitrate of bismuth to be taken several times a day. The patient reported to me afterwards that the treatment had given him great relief both in regard to the painfulness of the disease and the character of the discharges. Now this effect must have been produced by some impression on the nervous system, or else on the mucous membrane of the intestine that great "emunctory of the body." Dr. Miller says, "the most delicate chemical tests fail to detect evidence of its presence in the blood, in the urine, or in any secretion of the body." Bartholow says of the preparations of bismuth, "they are not entirely insoluble, for bismuth can be detected in the blood, urine and other secretions after a course of this medicine."

From my own observations with bismuth I think it has very little general effect, except it be indirectly, and when I have used it, whether internally or externally it has been chiefly for its local effect, occasionally as a gastric antacid.

That it is possessed of virtues as a local application I feel strongly persuaded. I have known the sensation of cooling and soothing as an effect attributed to it when applied to irritable ulcers and abraided surfaces. A young woman (school girl) from some cause not remembered had a round penetrating ulcer of the leg below the knee. Other things had been used without effect, when I directed it to be kept filled with bismuth; it commenced healing at once and in a short time was well.

It must be admitted, however, that up to this time its use in medicine has been more empirical than scientific. "New and more careful study of its effects is demanded, in which all possible sources of error or misrepresentation shall be avoided."

Dr. Moore's case was singular, but it is probable there was a twin pregnancy at first, and that one membrane being more fragile and easily ruptured than the other the contents were cast off while the other remained longer. When the threatening symptoms of abortion occar, it should not be taken for granted at once that abortion must occur. I have known many cases that have been restrained and the pregnancy kept on to full term ; one especially where there was much pain and smart hemorrhage of some days' continuance, was put back and resulted in the birth at full term of two fine boys.

Dr. West's case of hernia had a fortunate termination, but it is surprising how a knuckle of intestine could slough off and leave the canal pervious. I once had a case similar in some respects to this. It was a case of congenital hernia ; the subject was a young man just grown ; he had been in the habit of reducing it himself when it got in the way, but this time he could not; it became irreducible and strangulated. Being of the congenital kind it admitted of more delay. He had been in the hands of others for several days when I saw him. The hernia was found to be irreducible and I proceeded to operate. When I cut down to the sack and opened it (I always open the sack) the intestine was adhered to the sack and the sack strongly fixed to the surrounding tissues. The protruded parts, (omentum and knuckle of intestine) could not be returned without too much violence, so I approximated to some extent the edges of the wound and left the patient in the hands of one of his former attendants. In the course of a couple of days the attachments, weakened by sphacelation, gave way and allowed the intestine to slip back into the abdomen, the bowels sooon made a natural action, the outside omentum sloughed off, the wound healed, he soon made a good recovery and the hernia seems to be permanently cured. E. F. STARR, M. D.

Nacoochee, Ga., May, 1884.

